# The frequency of CD25+CD4+ and FOXP3+ regulatory T cells in ectopic endometrium and ectopic decidua

**DOI:** 10.1186/1477-7827-8-116

**Published:** 2010-10-05

**Authors:** Pawel Basta, Marcin Majka, Wojciech Jozwicki, Ewelina Lukaszewska, Anna Knafel, Marek Grabiec, Elzbieta Stasienko, Lukasz Wicherek

**Affiliations:** 1 Department of Gynecology and Oncology of the Jagiellonian University, Krakow, Poland; 2 Gynecology and Oncology Department of the Lukaszczyk Oncological Center and Chair of Gynecology, Oncology and Gynecological Nursing of the Ludwik Rydygier Collegium Medicum, 2 Romanowskiej Str, 85-796 Bydgoszcz, Poland; 3 Transplantology Department of the Jagiellonian University, Krakow, Poland; 4 Department of Tumor Pathology and Pathomorphology of the Ludwik Rydygier Collegium Medicum, Lukaszczyk Oncology Center, Bydgoszcz, Poland; 5 Department of Obstetrics, Gynecology and Oncological Gynecology of the Ludwik Rydygier Collegium Medicum, Bydgoszcz, Poland

## Abstract

**Background:**

The presence of regulatory T (Treg) cells in human endometrium is crucial for maintaining immunological homeostasis within the uterus. For this study we decided to evaluate the subpopulations of Treg cells in conditions where a disturbance in the immunological equilibrium in ectopic endometrium and decidua has been observed, such as in cases of ovarian endometriosis (involving local immune cell suppression) and ectopic pregnancy (involving an increase in local immune system activity). We then compared these findings to what we observed in the normal eutopic endometrium of women during the secretory phase of the menstrual cycle (with immune cells under individual control).

**Methods:**

The endometrium tissue samples evaluated in our study were obtained from 47 women during one of two kinds of laparoscopic procedures. 16 of the women underwent laparoscopies due to Fallopian tube pregnancies (EP), and 16 due to ovarian endometrioma, while 15 women made up a control group. The presence of regulatory T cells in these tissue samples was evaluated by FACS.

**Results:**

In our study, the percentages of FOXP3+ cells within the subpopulation of CD4+ T lymphocytes found in the decidua of the patients treated for Fallopian tube pregnancies were statistically significantly lower than both those observed in the ovarian endometriosis tissue samples and those found in the secretory eutopic endometrium samples of the control group.

**Conclusion:**

The disturbance in the immunological equilibrium observed in ectopic endometrium and decidua would seem to be related to the alteration in the Treg cell population that occurs in these ectopic tissues.

## Background

During the normal menstrual cycle the endometrium within the uterus is widely infiltrated by immune cells [1-3]. The specific activities of these immune cells are crucial for the proper course of such reproductive processes as menstruation and implantation. The suppressive profile of the endometrium is what enables the respective activities of these infiltrating immune cells. This suppressive phenomenon of the endometrium is a result both of the ability of endometrial cells to suppress immune cells and of the infiltration of the endometrium by such immune regulatory cells as regulatory T (Treg) cells or suppressive macrophages (B7H4) [4-6]. In some cases, however, the endometrium, which constitutes an active form of immunoregulatory tissue, is located outside the uterus. In other cases, as in ectopic pregnancy (e.g., within the Fallopian tube), the trophoblast cells may be located outside the uterus, even though the infiltrating trophoblast cells within the tubal wall are linked to the decidualization of epithelial and stromal cells. In cases of ovarian endometriosis and of ectopic pregnancy, mononuclear immune cells are recruited to the microenvironment of ectopic lesions; after this Treg cells infiltrate the tissue [7,8].

Treg cells are crucial for the tolerance and prevention of autoimmunity and have been described as pivotal both for the survival of allogeneic organ grafts and for the evasion of immune surveillance by fetal cells within the uterus [4-13]. Moreover, it has been observed that the number of Treg cells in the peripheral blood not only increases in women during pregnancy but also that their level of infiltration within the endometrium and decidua becomes elevated [11]. While Tilburgs *et al. *have shown that fetus-specific Treg cells are recruited to the decidua from the periphery [14], *Arruvito et al*. have demonstrated that the changes that take place in the Treg cell population in the peripheral blood over the different menstrual cycle phases are crucial for the proper course of reproductive processes. More specifically, the authors of the latter study found that the deregulation of the frequency of Treg cells in women with recurrent spontaneous abortion (RSA) may contribute to reproductive failure [15]. Recently, Schumacher *et al. *have shown that Treg cell attraction to decidua is associated with the high HCG concentration that occurs during intrauterine pregnancy development [16]. By contrast, the Treg cell concentration within the decidua derived from women with ectopic pregnancy or who suffer from spontaneous abortion is lower because the concentration level of beta HCG is lower in such conditions than it is in normal pregnancy [16]. Moreover, the infiltration level of Treg cells within decidua is decreased in patients suffering from spontaneous abortion compared with the level of infiltration found in the decidua of those undergoing induced abortions [9], and spontaneous abortion is strongly associated with an increase in the activity of the maternal immune system [17-19]. Furthermore, it has been shown that the rupture of the tubal wall is a result not only of trophoblast cell infiltration, but more importantly of the accumulation of immune cytotoxic cells within the ectopic ovum microenvironment [6]. In contrast to the situation with ectopic decidua in the Fallopian tube, local suppression of immune cytotoxic cells has been observed within ectopic endometrium (such as ovarian endometrioma) during the development of endometriosis [20]. Recently, Berbic *et al. *have demonstrated the presence of Treg cells within both the ectopic and eutopic endometrium of women suffering from peritoneal endometriosis. They showed that the FOXP3 expression within the eutopic endometrium derived from women with peritoneal endometriosis differs from that observed in healthy women [21]. An alteration in Treg lymphocyte infiltration generally disrupts the immunological equilibrium. Since the potential role of Treg cells in maintaining homeostasis in ectopic endometrium and decidua has not been precisely established in the literature, from a clinical standpoint, both endometriosis and ectopic pregnancy constitute unresolved problems. For this reason, we have chosen the specific type of ectopic endometrium and decidua found at the beginning of decidualization as a research model in order to demonstrate the potential role of Treg cells in the development of the suppressive profile of the endometrium. Thus to reiterate, the aim of our present study has been to evaluate the subpopulations of Treg cells in ectopic endometrium and decidua.

## Methods

### Patients

The endometrium tissue samples evaluated in our study were obtained from 47 women during one of two kinds of surgical procedures. 16 of the women underwent laparoscopic procedures because of unruptured Fallopian tube pregnancies (EP group, mean age 30.7 (± 6.1) years) and 16 because of ovarian endometrioma, (OE group, mean age 32.3 ± 5.2) while 15 (constituting the control group, mean age 35.5 ± 8.1) underwent curettage as an additional procedure during uterine cervix biopsy on account of a diagnosis of cervical intraepithelial neoplasia or CIN (it should be noted that the biopsy of the endometrium was an additional procedure for which the patient's consent was obtained).

The ectopic endometrium tissue samples were derived from the women who underwent laparoscopy due to ovarian endometrioma while the ectopic decidua samples were derived from those patients who underwent the procedure because of ectopic pregnancy. Meanwhile, the eutopic endometrium tissue samples were derived from the patients on whom curettage was performed. The patients included in the study were treated between January 2007 and June 2010 in the Department of Gynecology and Oncology at the Jagiellonian University, Krakow, Poland. The patients referred to the control group were randomly selected from the group who had curettage following uterine cervix biopsy because they were diagnosed with cervical intraepithelial neoplasia. The patients from the control group were not found to have any other pathological lesions within the reproductive tract and had normal, regular menstrual periods.

Patients with intrauterine infections (as confirmed by histopathological examination) were excluded from the study. Patients with concomitant systemic diseases, diseases of the thyroid gland, diabetes or hypertension, or the presence of any other lesions as detected by gynecological, ultrasound, cytology, or colposcopy examinations were also excluded. Furthermore, none of the patients included in our study received any hormonal treatment. The tissue samples were then independently evaluated using routinely stained (hematoxylin and eosin) slides prepared from paraffin-embedded tissue material, enabling accurate diagnosis: the presence of ectopic pregnancy or ovarian endometrioma was confirmed in each of the patients. All of the patients were then classified into subgroups according to the particular menstrual cycle phase as determined by the histopathological analysis of the tissue samples obtained during surgery and the results of clinical examination together with an assessment of the levels of progesterone, estradiol, and follicle-stimulating hormone (FSH) in the blood sera. (Responsible OE group- 8 patients during proliferative cycle phase FSH mlU/ml 5.62 (± 2.4) mlU/ml; estradiol 140.1 (± 34.2) pg/ml; progesterone 0.87 (± 1.9) ng/ml; OE group-8 patients during secretory cycle phases FSH 6.5 (± 4.1) mlU/ml; estradiol 159.3 (± 66.3) pg/ml; progesterone 6.4 (± 3.2) ng/ml; Control group FSH 8.2 (± 5.13) mlU/ml; estradiol 131.4 (± 53.2) pg/ml, progesterone 7.56 (± 2.1) ng/ml.).

The patient's consent was obtained in each case. Prior to the present study we also obtained the approval of the Jagiellonian University Ethical Committee for our research program (DK/KB/CM/0031/447/2010, KBET/135/B/2007).

### Decidual mononuclear cell isolation

The decidua and endometrium samples were cut into small fragments and disintegrated by being smashed through a 40 μm cell-strainer. The realized cells were then spun down and the resulting pellet was subjected to ammonium chloride lysis in order to get rid of any contaminating red blood cells. Following lysis, the cells were washed in PBS. They were then re-suspended in PBS, counted, and used either immediately for staining or frozen for future analysis (cells were frozen in a medium consisting of10% DMSO, 5% albumin, and 85% of a cell culture medium).

### Flow cytometry

**Figure 1:**
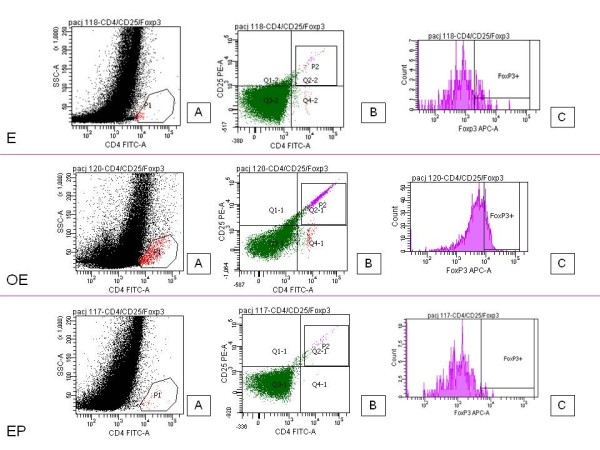
**Treg cell gating strategy**. The characterization of the subpopulation of Treg cells (CD4+CD25+FOXP3+) within the subpopulation of T lymphocytes in eutopic endometrium during the secretory cycle phases (E), ovarian endometriosis (OE), and Fallopian tube pregnancy (EP). Left panel shows the gating strategy for CD4-positive cells (P1). Middle panel is used to set the gate on double positive CD4^+^CD25^+ ^cells (P2). On the right panel the histogram representing FoxP3 staining is shown (FoxP3^+^).

The cell phenotype was analyzed with the panel of mAb-CD4 FITC, CD25 APC, and FOXP3 PE (Pharmingen). Briefly stated, to the 1 × 10^6 ^cells suspended in 60 μl of staining buffer (PBS, 2% FBS) 20 μl of each mAb (CD4, CD25) was added. Next the cells were incubated in the dark for 30 min at 4°C. After incubation the cells were washed twice in PBS and were permeabilized with FoxP3 permeabilization buffer (Becton Dickinson; USA) for 10 min at room temperature in the dark. Following this, they were stained with anti-FOXP3 antibodies for 30 min at 4°C in the dark. The stained cells were then washed and collected using the FACSC anto-cytometer (Becton Dickinson; USA), and finally were analyzed with FACS Diva software (Becton Dickinson; USA). Each time 3 × 10^4 ^events were saved for analysis. Logical gates were used to analyze particular populations of cells. The cells were gated first according to SSC and CD4FITC parameters (gate P1). After this, the cells from gate P1 were analyzed according to CD25PE and CD4FITC parameters, and gate P2 was established on double positive CD4FITC CD25PE cells. The double positive cells from gate P2 were analyzed for the presence of FoxP3 antigen. The gate FoxP3+ was set on cells which had stained positively with FoxP3 APC antibodies (Figure 1).

### Statistical analysis

The statistical significance of the recorded differences among the groups was determined by the Kruskal-Wallis analysis of variance (ANOVA). All statistical analyses were carried out with the Statistica 8.0 software program (StatSoft Inc., Tulsa, OK, USA). A p value < 0.05 was considered indicative of statistical significance.

## Results

CD25+CD4+FOXP3+ T cells were found in all the examined endometriosis tissue samples. These same cells were observed in 72% of the eutopic endometrium tissue samples but were present in only 29% of the tissue samples taken from patients who had ectopic pregnancies (Figure 1).

We did not observe any differences over the course of the menstrual cycle in the percentage of FOXP3+ positive cells within the subpopulation of CD4+T lymphocytes in ectopic endometrium in the tissue samples from the group of patients with ovarian endometriosis. The median for OE during the proliferative and secretory cycle phases were 8.5 (16.7) and 6.3 (5.9) respectively.

**Figure 2:**
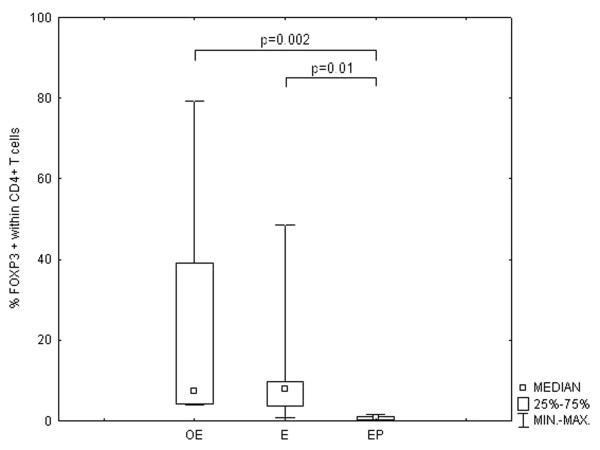
**The changes in the percentage of FOXP3+ cells within the subpopulation of CD4+ lymphocytes**. The comparison of the percentages of FOXP3+ cells within the subpopulation of CD4+ lymphocytes in the ectopic endometrium (OE) tissue samples derived from patients with ovarian endometriosis and in ectopic decidua (EP) derived from patients with Fallopian tube pregnancy with the percentages found in the eutopic endometrium samples derived from patients during the secretory cycle phases (E). The data is presented as a median ± IQR (Intraquartile Range).

The percentage of FOXP3+ cells within the subpopulation of CD4+ lymphocytes was statistically significantly higher in the tissue samples from the patients with endometriosis as compared with the percentage found in the tissue samples from the patients who had developed ectopic pregnancies. Likewise, the percentage of FOXP3+ cells within the subpopulation of CD4+ lymphocytes was higher than in the endometrium from the control group, but these differences were not statistically significant. The percentages of these cells observed in the endometrium from the control group were, however, statistically significantly higher than those found in the ectopic deciduas (Figure 2).

**Figure 3:**
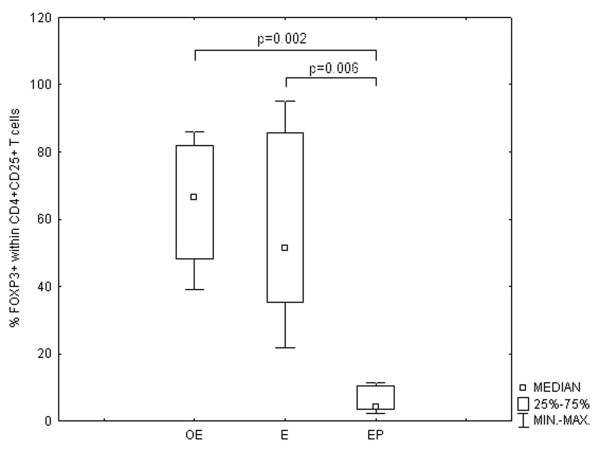
**The changes in the percentage of FOXP3+ cells within the subpopulation of CD4+CD25+ lymphocytes**. The comparison of the percentages of FOXP3+ cells within the subpopulation of CD4+CD25+ lymphocytes in the ectopic endometrium (OE) tissue samples derived from patients with ovarian endometriosis and in ectopic decidua (EP) derived from patients with Fallopian tube pregnancy with the percentages found in the eutopic endometrium samples derived from patients during the secretory cycle phases (E).The data is presented as a median ± IQR (Intraquartile Range).

Similar results were obtained from analysing FOXP3 positive cells within the subpopulation of CD4+CD25+ lymphocytes (Figure 3).

## Discussion

In our study, the percentages of FOXP3+ cells in the subpopulation of CD4+ T lymphocytes found in the decidua of the patients treated for Fallopian tube pregnancy were statistically significantly lower than both those observed in the ovarian endometriosis samples and those found in the secretory eutopic endometrium of the control group.

Single statements derived from the literature on the subject indicate that Treg lymphocytes are present within ovarian endometrial tissue [8,21]. Furthermore, it has been demonstrated that the percentage of FOXP3 positive lymphocytes found within the subpopulation of T cells in the peripheral blood of women suffering from ovarian endometriosis does not differ from that observed in healthy women [8]. Berbic *et al. *previously demonstrated the presence of Treg cells within peritoneal ectopic endometrium [21]. In that study the authors demonstrated that there was an alteration in the FOXP3 expression within the eutopic endometrium that was derived from women suffering from peritoneal endometriosis during the secretory cycle phases when compared to what was observed in the tissue taken from healthy women [21]. For fertile women the significant decrease in the Treg cell population in the peripheral blood typically occurs just after ovulation [15]. By contrast, the women suffering from recurrent spontaneous abortion (RSA) were characterized by an absence of fluctuation in the number of Treg cells [15]. The increase in the number of Treg cells depends upon estrogen levels [22], and estrogens are also responsible for the increase in the immune suppressive potential of these cells [22]. Estrogen levels generally seem to be linked with a decrease in TH1 response [22]. Furthermore, a local increase in estrogen levels is characteristic of patients with ovarian endometrioma lesions. In our study the percentages of FOXP3+ cells in the subpopulation of CD4+ T lymphocytes found in eutopic endometrium were slightly lower than those found in ovarian endometrioma tissue. Additionally, no differences in the percentage of Treg cells within the T lymphocyte subpopulation were observed over the course of the menstrual cycle in the ovarian endometriosis tissue samples. Most likely, the absence of Treg cell fluctuation can be linked to an immune defect arising with the development of endometriosis. Although in 95% of cases the endometrial cells migrate along with retrograde menstruation from the endometrial cavity to the peritoneal cavity, only 15% of them actually survive in ectopic localization despite the host immune system response [23]. Since they are able to regulate the activity of immune cells, such cells themselves evade immune surveillance. The immunomodulatory activity of endometrial cells is crucial for the proper course of reproductive processes. Under normal physiological conditions the intensity of the immune suppressive activity of the endometrium depends on the menstrual cycle phase and differs significantly between the proliferative and secretory phases (as has been observed by Arruvito *et al. *in a study on the percentage of Treg cells in the late proliferative and early secretory cycle phases [15] or in different studies concerning the expression of the factors responsible for immune cell suppression, such as RCAS1 expression within endometrial cells) [24]. In patients with endometriosis, however, the suppressive activity of the endometrium does not fluctuate. In our previous studies we have demonstrated that both the expression of RCAS1 and HLA-G in ovarian endometriosis and its concentration in the peripheral blood does not differ between the proliferative and secretory cycle phases [25,26]. Both protein RCAS1 and HLA-G are important immunosuppressive factors [27-30]. The suppressive profile of the endometrium therefore depends not only on the absence of physiological changes--as has been observed from the Treg cell population--but also on the over-expression of suppressive factors within ectopic endometrium. For example, the ovarian endometriosis tissue samples were characterized by an over-expression of mRNA for FOXP3 in comparison to normal endometrial tissue [8]. Additionally, women experiencing primary unexplained infertility have significantly lower FOXP 3 expression within the endometrium than do fertile women [31]. These studies thus show how important the proper regulation of immune cell activities is for maintaining homeostasis during pregnancy. Sasaki *et al. *have reported that about 7% of CD4+ cells in the decidua derived from patients suffering spontaneous abortion are CD4+CD25^bright ^cells and that this percentage is lower than in the tissue of women experiencing normal pregnancies [9]. Furthermore, Shansham *et al. *have shown that patients suffering unexplained spontaneous abortion were typified by a smaller proportion of CD4+CD25+FOXP3+ lymphocytes within decidua than women experiencing normal pregnancies. Recently, Arruvito *et al *have demonstrated that the Treg lymphoctes of women suffering RSA typically exhibit lower than normal suppressive activity [32]. Thus the decrease in the level of Treg cell infiltration within decidua may result in an increase in both cytotoxic T lymphocyte and NK cell activity. During pregnancy Treg cells are recruited to the decidua [10], but the percentage of Treg cells in both the decidua and the peripheral blood increases only until spontaneous labor begins [14]. When recruited to the endometrium, Treg cells are directly able to suppress the immune response to both fetus-specific and fetus non-specific antigens [14,33]. The beginning of spontaneous labor, however, is associated with a decrease in the percentage of Treg cells within decidua along with a subsequent increase in the immune response [34-37], as has also been observed during spontaneous abortion [17]. The activity of immune cells within the uterus is precisely controlled by many factors, and the appearance of such pathology as preeclampsia, abortion, placental abruption, and retained placental tissue results from a disturbance in the regulation of maternal immune cells [38-40]. Just such a situation has also been observed during the development of ectopic Fallopian tube pregnancy where the tubal rupture is seen as a consequence of the increase in maternal immune cell activity [6,41]. In our previous study we have demonstrated that the development of Fallopian tube pregnancy is related both to the accumulation of cytotoxic immune cells and NK cells within ectopic decidua and to a continued increase in the activity of these cells within the tubal wall [6,41]. In our current study, however, we observed that the Treg cell accumulation within the tubal wall was not as dense as it was within the eutopic endometrium during the secretory cycle phase. This is not unlike what occurs in eutopic endometrium with Arias Stella reaction where the Treg cell population decreases in comparison to that of eutopic endometrium during the secretory cycle phases [42]. The decrease in the accumulation of Treg cells in ectopic decidua within the Fallopian tube wall observed both in the Schumacher *et al. *[16] study and in our current one would therefore seem to be associated with an increase in the activity of the immune cells infiltrating these tissues that leads finally to tubal rupture. This contrasts with the conditions linked to ectopic endometrium; ovarian endometrioma development, for example, is associated with an immune defect. This observation would seem to accord with our own results showing that the percentage of the Treg cells within the overall cell population was the highest in the ovarian endometrioma tissue samples.

The proper balance between immune cell activity and the intensity of the suppressive profile of the endometrium or decidua is what enables the proper course of physiological reproductive processes. The correct balance is a result of the accumulation of Treg lymphocytes within the endometrium and decidua respectively. Such processes as the development of ectopic pregnancy and ovarian endometrioma are associated with a disturbance in the suppressive profile of the endometrium resulting from the alteration in the Treg cell population.

## Conclusion

The disturbance in the immunological equilibrium observed in ectopic endometrium and decidua is associated with the alteration in the Treg cell population that occurs in these ectopic tissues.

## Competing interests

The authors declare that they have no competing interests.

## Authors' contributions

PB conceived of and designed the study, analyzed and interpreted the data, and drafted the manuscript. LW managed the realization of the project; he also helped to conceive of and design the study as well as draft the manuscript. MM carried out the molecular study and revised it. EL was also involved in carrying out the molecular study. MG, AK, ES and WJ all participated in the final revision of the manuscript. All the authors read and approved the final manuscript.
